# A Lightweight Pose Sensing Scheme for Contactless Abnormal Gait Behavior Measurement

**DOI:** 10.3390/s22114070

**Published:** 2022-05-27

**Authors:** Yuliang Zhao, Jian Li, Xiaoai Wang, Fan Liu, Peng Shan, Lianjiang Li, Qiang Fu

**Affiliations:** 1 Sensor and Big Data Laboratory, Northeastern University, Qinhuangdao 066000, China; 2172080@stu.neu.edu.cn (J.L.); 2101929@stu.neu.edu.cn (X.W.); peng.shan@neuq.edu.cn (P.S.); lilianjiang@live.cn (L.L.); 2 Hebei Key Laboratory of Micro-Nano Precision Optical Sensing and Measurement Technology, Qinhuangdao 066000, China; 3 College of Life Science and Technology, Xi’an Jiaotong University, Xi’an 710049, China; 2213722832@stu.xjtu.edu.cn; 4 Shijiazhuang School, People Liberation Army Engineering University—Shijiazhuang, Shijiazhuang 050003, China; fuq2124@gmail.com

**Keywords:** abnormal gait behavior, OpenPose, machine learning, XGBoost, random forest

## Abstract

The recognition of abnormal gait behavior is important in the field of motion assessment and disease diagnosis. Currently, abnormal gait behavior is primarily recognized by pressure and inertial data obtained from wearable sensors. However, the data drift and wearing difficulties for patients have impeded the application of these wearable sensors. Here, we propose a contactless abnormal gait behavior recognition method that captures human pose data using a monocular camera. A lightweight OpenPose (OP) model is generated with Depthwise Separable Convolution to recognize joint points and extract their coordinates during walking in real time. For the walking data errors extracted in the 2D plane, a 3D reconstruction is performed on the walking data, and a total of 11 types of abnormal gait features are extracted by the OP model. Finally, the XGBoost algorithm is used for feature screening. The final experimental results show that the Random Forest (RF) algorithm in combination with 3D features delivers the highest precision (92.13%) for abnormal gait behavior recognition. The proposed scheme overcomes the data drift of inertial sensors and sensor wearing challenges in the elderly while reducing the hardware requirements for model deployment. With excellent real-time and contactless capabilities, the scheme is expected to enjoy a wide range of applications in the field of abnormal gait measurement.

## 1. Introduction

Abnormal gait behavior is highly-related to many neurodegenerative diseases, such as Parkinson’s disease, cerebral palsy, lumbar disc herniation, cerebral infarction and osteoarthritis. Therefore, the recognition and measurement of abnormal gait behavior has been an important topic of research in the field of diagnosis and treatment [1]. Abnormal gait behavior is highly prevalent, especially in the elderly. According to the statistics of the China Parkinson’s Disease Registry (CPDR), more than 3 million patients suffer from Parkinson’s symptoms in China. This indicates an urgent need for recognition systems for behavioral disorders [2]. Preliminary diagnosis of a patient’s disease based on their abnormal gait behavior is needed in many everyday life settings, such as houses, nursing homes, and other public places, which saves much cost and time for the patient. At present, the abnormal gait behavior of patients can be recognized by two main categories of methods: by using inertial measurement units (IMUs) and by using contactless models and machine learning algorithms.

A preliminary diagnosis of abnormal gait behavior can be achieved by wearing micro sensors. SIJOBERT et al. [3] extracted features from frozen gait by placing a wireless inertial sensor on the patient’s lower leg to acquire changes in gait parameters. Zhao et al. [3,4] developed a gait analysis system consisting of a bipedal IMU. By using an inequality-constrained zero-velocity update (ZUPT) aided INS algorithm, this system provides an efficient method for estimating gait parameters and characterizing gait performance to assess the rehabilitation process of patients with gait disorders. Wang et al. [5] developed a new IMU-based clinical gait assessment method. Their experiment extracted nine variables from two calf-mounted IMUs and used them to quantify the patient’s gait deviation. Based on these parameters, an IMU-based gait normal index (INI) was derived to assess the overall gait performance. However, the use of sensors to recognize abnormal gait behavior in patients with mobility impairments suffers from data drift problems and wearing difficulties [5].

In recent years, inertial and pressure sensors have been widely used in hospitals and nursing homes for analyzing patients’ gait [6]. However, such methods are suitable for patients who have difficulty wearing sensors for data acquisition. Therefore, it is necessary to explore contactless systems for diagnosing different behavioral disorders [7]. Kursun et al. [2] proposed a method that combines the support vector machine (SVM) algorithm and a recognition model for the preliminary diagnosis of patients with Parkinson’s symptoms. Using acoustic data with the smallest deviation, the method can distinguish patients with Parkinson’s disease from healthy people at an accuracy of 92.75%. Yaman et al. [1] found through experiments that patients with Parkinson’s disease have poor verbal ability, so they proposed a method in which SVM and k-nearest neighbors (KNN) algorithms are used to obtain features from the Parkinson’s acoustic data set for the recognition of Parkinson’s disease. The accuracy was calculated to be 91.25% and 92.33%, respectively, by using the two algorithms. Sato et al. [8] obtained frozen gait and Magnetic step data of Parkinson’s patients by using OP forward gait features. By analyzing the data curve, they found patients with Parkinson’s disease have a different movement curve from healthy people. Liu et al. [9] proposed a locally weighted discriminant-preserving projection embedding ensemble algorithm to solve the problems of high noise and small sample size with Parkinson’s disease data. The algorithm achieved improved accuracy in Parkinson’s disease recognition. Studies have found that contactless methods can better differentiate patients with Parkinson’s disease and healthy people.

However, there is still a lack of studies on the recognition of gait behavior differences caused by diseases such as cerebral infarction, cervical compression, cerebellar lesions, and lumbar disc herniation. When it comes to diagnosing a patient, it is necessary for the doctor to first make a preliminary diagnosis of the type of disease that causes the abnormal gait behavior.

Guo et al. [10] went a further step by using an OP model to assess six abnormal toe types with a mobile 3D gait analysis system. Later, D’Antonio et al. [11] solved the problem of information concealment in videos with a corrected OP model. They also used an IMU sensor to calibrate the collected data, which verified the authenticity of features extracted by the OP model. At present, SVM and KNN are among the mainstream algorithms for the recognition of behavioral disorders. Chen et al. [12] used a new FKNN model to classify the Parkinson’s data set and achieved an experimental accuracy of 96.07%. Hariharan et al. [13] adopted a feature reduction/selection technique and a recognition algorithm to detect Parkinson’s symptoms. The recognition process was performed using least squares SVM (LS-SVM), probabilistic neural network (PNN), and general regression neural network (GRNN), and the recognition accuracy was as high as 100%.

To sum up, most of these techniques emphasized the recognition accuracy of abnormal gait behavior over the recognition efficiency. This means challenges remain to deploy these techniques in devices in daily applications. When a traditional OP model is used, in particular, it can be difficult to achieve real-time disease identification without the support of powerful hardware. Therefore, we developed a novel method that can recognize abnormal gait behavior accurately and efficiently. First, an ultra-lightweight OP model was developed to enable much-increased efficiency at the price of a little bit lower accuracy. Then, based on the gait features obtained using the OP model, a simple 3D reconstruction model was developed to supplement more accurate features. At last, some highly efficient machine learning algorithms were used to recognize abnormal gait behavior. Our scheme achieves a contactless recognition of abnormal gait behavior due to multiple types of diseases compared to previous work.

Here is a summary of what we did and accomplished in this work:

1. We constructed a lightweight OP model with Depthwise Separable Convolution for real-time extraction of abnormal gait features. This significantly reduced the computing workload required for hardware-intensive devices.
2. We performed a 3D reconstruction on the 2D lower limb data extracted from subjects and obtained a total of 11 abnormal gait features from that data. Then, we further processed the extracted data to obtain step length features. These steps improved the data structure and diversified feature types.
3. We used machine learning algorithms to filter and classify abnormal gait features to the measurement of abnormal gait behavior caused by different diseases.

## 2. Experimental Method

### 2.1. Establishment of Experimental Models

Usually, the lower limb behavior of the human body is captured by a lightweight OP model, which offers a quick solution to process video and image data in real time [11,14].

**Figure 1:**
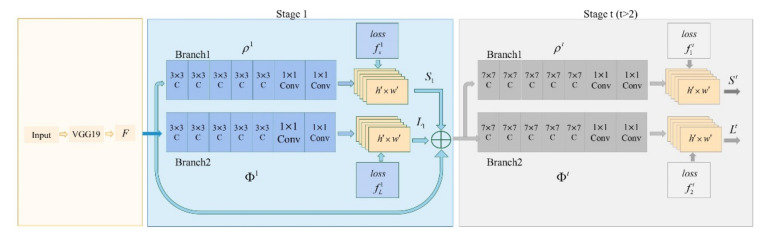
Structure of the OP model.

Our work used this model to identify the 2D joint coordinates of patients during walking and to obtain their walking pose data by extracting the coordinates of their lower limb joints [15,16]. This vision-based model eliminates the inertial drift problem with traditional sensors, and its structure is illustrated in Figure 1. With the image stream data to be processed by the OP model, the feature map *F* was obtained through the VGG19 network. Then, the data entered the dual-branch convolutional neural network in multiple stages through the feature map *F*. The upper branch was used to predict the heat map of the joint, which was obtained as the heat map S. The lower branch was used to predict the affinity field of the joint. Each stage was further predicted, and finally, the joint heat map and affinity field of the entire network were obtained after t times of recognition [14].

In Figure 1, $\rho^{t}$ and $\varphi^{t}$ are convolutional neural networks used to read features in stage t to generate a joint heat map $S^{1}=\rho^{1}\left(F\right)$ and joint affinity field $L^{1}=\varphi^{1}\left(F\right)$; $\rho^{1},\rho^{t},\varphi^{1},\varphi^{t}$ were composed of five convolution blocks and two 1 × 1 ones. The input for each stage was the image feature *F* and the recognition result of the previous stage. $S^{t},L^{t}$ are the heat map and affinity field of joint at stage t, respectively. Then, the convolutional network of this stage was used to predict the joint heat map and joint affinity field of this stage. The recognition process can be expressed as follows:

(1) $$S^{t}=\rho^{t}(F,S^{t-1},L^{t-1}),\forall t\geq 2$$

(2) $$L^{t}=\varphi^{t}(F,S^{t-1},L^{t-1})$$

To obtain the coordinates of the lower limbs for real-time gait recognition, a Depthwise Separable Convolution structure, instead of the conventional convolution in VGG19, was used in our experiment. This can significantly reduce the number of model parameters required [17]. The size of all convolution kernels was set to 3 × 3, and the number of convolution kernels increased with the number of layers. The Depthwise Separable Convolution used different convolution kernels to convolve different channels, and decomposed the ordinary convolution into two processes: Depthwise Convolution and Pointwise Convolution, so as to decouple channel correlation and spatial correlation [17]. The Depthwise Convolution process split the convolution kernel into single channels and convolved each channel without changing the depth of the input feature image. The Pointwise Convolution process was used to up- and down-dimension the feature map with 1 × 1 convolution. The combination of these two processes made the model more lightweight. N conventional convolution kernels of size *D_K_* × *D_K_* × *M* were equivalent to one Depthwise Convolution and N Pointwise Convolutions. Therefore, the FLOPS and Params of the Depthwise Separable Convolution were reduced to *(1/N) + (1/* *D_K_^2^)* conventional convolutions. Since there were 16 convolutions of size 3 × 3 in VGG-19, the FLOPS and Params of the lightweight OP model dropped to 17.36% of the original model.

A convolutional neural network with a smaller size and less computation was formed, which was well-suited for mobile devices and enabled faster and more efficient extraction of features from video stream data and reduced hardware requirements for model deployment.

### 2.2. 3D Construction of Lower Limbs

**Figure 2:**
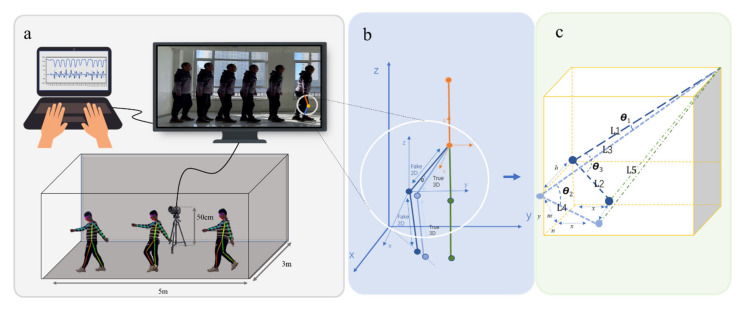
(**a**) Data acquisition process. (**b**) The 3D spatial relationship between the real knee and the mapped knee. (**c**) The lower limb reconstructed through the 2D data.

During data acquisition, the camera was located in the middle of the walking distance of the person, at a distance of 3 m from the vertical position of the person. There was a smaller angle between the video of the person during walking and the position of the camera, as shown in Figure 2a. The computer displayed the knee angle in motion, the velocity of the knee angle variation, and the acceleration of the knee angle variation. The positions of the thighs, calves and feet in the video were mapped to a two-dimensional (2D) plane. Therefore, errors were present in the length and angle data mapped in the video, and traditional IMU sensors have demonstrated errors in the knee angle measured by the OP [11,16,17]. As shown in Figure 2b,c, in the 3D space, there was an angle $\theta_{1}$ error between the mapped thigh and the real thigh. The real knee angle is represented by $\theta_{2}$, and the false knee angle of the mapped surface is represented by $\theta_{3}$. 

Since the data output from the OP model was 2D data, the angle data output by the leg needed to be reconstructed, and the reconstruction process is shown in Figure 2c. We obtained all the position coordinates of the leg joints and generated length and angle data by connecting the positions of the joint points. In the 2D image, $L_{1}$, $L_{2}$ and $L_{5}$ denote length data directly output by the OP model as extractable quantities, while $L_{3}$ and $L_{4}$ denote the real leg lengths in space. In the experiment, the data of the person standing in the video was used as the real leg length data. Finally, the 3D knee angle $\theta_{2}$ was obtained as follows:

(3) $$\cos\theta_{2}=\frac{L_{1}^{2}-L_{5}^{2}+L_{2}^{2}+2\sqrt{L_{3}^{2}-L_{1}^{2}}\times \sqrt{L_{4}^{2}-L_{2}^{2}}}{2L_{3}\times L_{4}}$$

## 2.3. Extraction of Step Length Features

**Figure 3:**
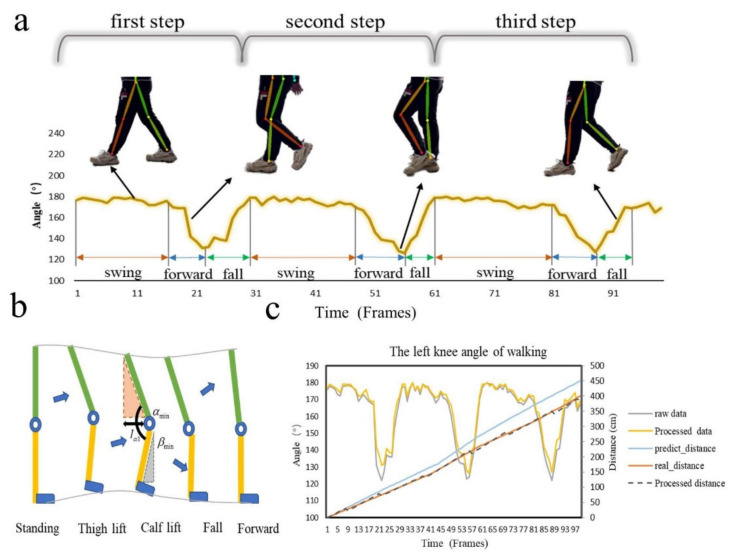
(**a**) The lightweight OP model captures the phases of the knee angle change during walking. (**b**) Step length calculation process. (**c**) Step length correction process.

Traditional gait features include multidimensional features such as step length, average stride time, average pace time, average stride length, and the lowest knee angle [6,18]. With the OP extraction model, we obtained abnormal gait features directly by intercepting each gait cycle in the program. In addition, the left and right step length features needed to be obtained by further processing the extracted data. Therefore, we designed an experiment for step length data extraction by observing the walking pose of the subject, as shown in Figure 3.

The human walking process mainly consists of forward, swing and fall, as shown in Figure 3a. Patients with gait behavioral disorders generally walk with left and right swings and rapid changes in step length. Therefore, step length data was extracted to serve as the predictive features for the subsequent experiments.

The step length feature extraction process is shown in Figure 3b. The distance for which a person walks one step with one foot is determined by the person’s leg length and knee angle during walking [19]. When a person leans forward, the raised foot is affected by the bending angle of the knee and moves forward. Taking the fixed angle of the step length as the lowest knee angle, we designed an experiment to measure the walking distance of a single person. We denoted the length of the thigh as $\Gamma_{1}$, the length of the calf as $\Gamma_{2}$, the lowest knee angle as $\alpha_{\min}$, and the angle between the calf and the vertical direction of the knee at the lowest knee angle as $\beta_{\min}$.

When the step length was completely determined by thigh length $\Gamma_{1}$ and lowest knee angle $\alpha_{\min}$, we obtained:

(4) $$l_{\alpha 1}=\Gamma_{1}\times \cos\left(\alpha_{\min}-90\right)$$

$l_{\alpha 1}$ represents the predicted real step. The predicted distances in Figure 3c represent the time-varying movement distance curve fitted by this step method. As time went by, the difference between the predicted real distance and the real value became larger. Therefore, it was necessary to process the experimental data. It was found that the position coordinate of the knee at the lowest angle did not accurately reflect the distance moved by a single step during the real walking process.

When the step length was determined by thigh and calf lengths $\Gamma_{1}$, $\Gamma_{2}$ and knee angles $\alpha_{\min}$ and $\beta_{\min}$, we obtained:

(5) $$l_{\alpha 2}=\Gamma_{1}\times \cos\left(\alpha_{\min}-90\right)-\Gamma_{2}\times \sin\left(\beta_{\min}\right)$$

The processed single-step step length data is also presented in Figure 3c. This data was close to the real data. This demonstrated that this contactless step length measurement method is scientifically feasible. In the experiment, left and right step lengths were used as the features for classifying different types of abnormal gait behavior.

## 3. Analysis of Abnormal Gait Behavior

### 3.1. Analysis of Gait Characteristics for Different Diseases

In the medical field, behavioral disorders are mostly diagnosed in patients with Parkinson’s disease, lumbar disc herniation, cerebral infarction, diabetes mellitus, and cerebellar lesions. Stimulation of electrical muscle signals can cause abnormal gait when walking. Therefore, there is a need to classify and assess these patients’ disorders in a quantitative and contactless manner. In our experiments, we extracted gait data by asking the subject to walk for a distance under an indoor camera. Then, using machine learning algorithms, we achieved a preliminary diagnosis of these diseases.

Table 1 lists five different abnormal gaits that may be caused by behavioral disorders and their characteristics. These characteristics can be used as the motor characteristics of subjects who showed such abnormal behavioral symptoms in the experiment. Five types of abnormal gait behaviors are caused by different types of diseases. When the walking stride is small and the movement is stiff and slow, it is a manifestation of Parkinson’s disease. Therefore, this scheme is convenient for doctors to pre-diagnose patients by classifying abnormal gait.

### 3.2. Collection of Experimental Data

Due to sensitive neuromuscular changes, patient gait can serve as an important tool for patient state prediction and classification, widely affecting most gait features such as knee angle, step size, and stride length [24,25]. Exploring the gait changes caused by the muscles caused by lesions can help to understand the gait changes and the rehabilitation process of various diseases [26].

**Figure 4:**
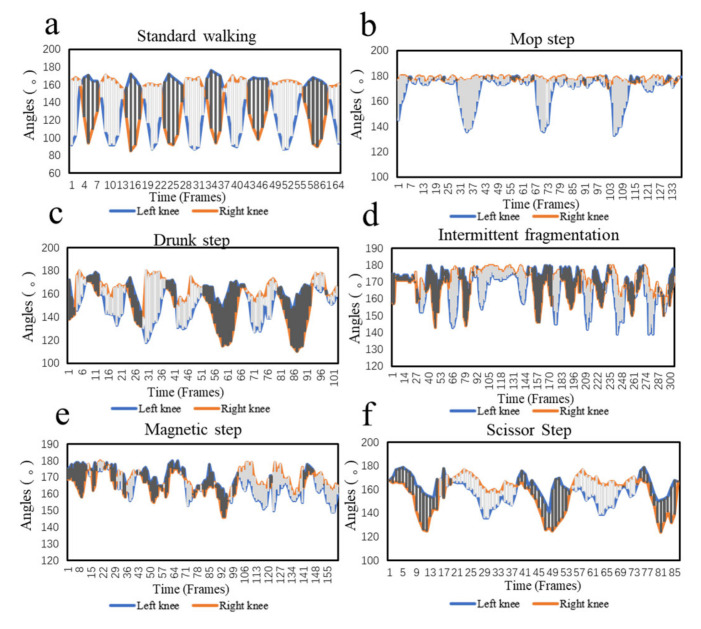
Variation curves of left and right knee angles under different gait. (**a**) Standard walking. (**b**) Mop step. (**c**) Drunk step. (**d**) Intermittent fragmentation. (**e**) Magnetic step. (**f**) Scissor step.

A total of eight subjects of different heights and weights (five males and three females) were involved in this experiment for data collection. Based on the behavioral characteristics of the disease in Table 1, the subjects were asked to imitate walking with a normal gait and five abnormal gaits. All the subjects walked back and forth along a five-meter-long experimental route. Five sets of experimental data were collected from each subject for each gait. Through the method of data undersampling, the imbalanced data set becomes balanced, and 40 experimental data are saved for each type of feature. The knee angle changes as the subjects walked with different gaits are shown in Figure 4. During the experiment, the subjects showed significant differences in knee angle changes among the six gaits. The changes in the left and right knee angles during walking with abnormal gaits suggested that the body was unbalanced.

### 3.3. Feature Screening

**Figure 5:**
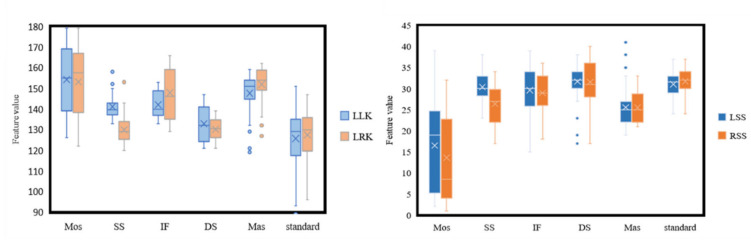
Difference distribution statistics of six gaits. (Features included are LLK, LRK, LSS, RSS).

We set one step for each left and right leg as a motion cycle. By collecting and processing the raw data, we obtained a total of 11 gait features, including left step length (LSS), right step length (RSS), lowest left knee angle (LLK), lowest right knee angle (LRK), average stride length (AS), average pace time (APT), average stride time (AST), variance of right knee angle variation (VOR), variance of left knee angle variation (VOL), average value of knee angular velocity (KAV), and average value of knee angular acceleration (KAA). We performed a 3D reconstruction on four of these features: LSS, RSS, LLK, and LRK. The four feature statistics of the six gaits with large differences are shown in Figure 5. Through statistical data, it is found that there are large differences in the features of different gait types, which contributes to higher accuracy of classification.

**Figure 6:**
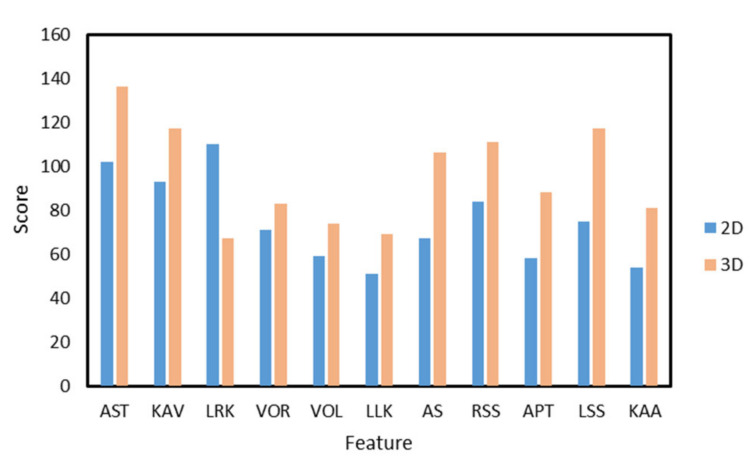
2D and 3D feature importance scores after XGBoost screening.

Admittedly, accidental errors and interfering characteristics were present in this experimental data. Therefore, before classifying abnormal gait behavior, we screened the feature data and obtained the importance scores of each feature using the XGBoost algorithm [27], as shown in Figure 6. (After GridSearch, it is determined that the parameter combination is booster is gbtree, the learning rate is 0.3, tree depth is 6, and the maximum number of iterations is 100).

From Figure 5, five of the eleven features, i.e., AST, KAV, APT, RSS, and LSS, have relatively high importance scores in both 2D and 3D conditions. For the four features processed with 3D reconstruction, the models showed increased importance scores on LSS, RSS, LLK and a decreased importance score on LRK. For models containing 3D features, AST achieved the highest importance score. For models containing 2D features, LRK achieved the highest importance score. The experimental results showed that the overall model performance varied greatly with the type of feature used in the experiment. Some features produced low importance scores, indicating that these features did not contribute much to the overall model performance due to the masking problem and the randomness of different subjects during data collection. Better results were achieved for AST and KAV than for the other features, indicating that the average stride time and knee angular velocity played a bigger role in assessing body balance during walking.

XGBoost is used as a key machine learning algorithm for feature importance ranking, which can eliminate unfavorable features of machine learning models [27,28,29]. As shown in Table 2, by reducing the low-scoring features in order of importance scores, we obtained the acceptance scores for different numbers of features. With 3D features, the acceptance scores decreased as the number of features decreased in the range of 1~8. The best score of 0.9306 was achieved with 11 or 8 features. For the 2D features, the best score was achieved with 11 features, and the score basically decreased as the number of features decreased. Since the same score was obtained with 8 or 11 3D gait features, the abnormal gait behavior is recognized with 8 and 11 features, respectively.

## 4. Discussion

The five abnormal gaits were mainly determined by different types of gait behavioral disorders. In our experiment, we used five recognition methods, i.e., Gradient Boosting (GB), KNeighbors (KN), Multilayer Perception (MLP), Random Forest (RF), and SVM, to classify the six gait features [28,29,30,31,32,33]. The parameter settings of the machine learning model obtained by GridSearch are shown in Table 3. Finally, 2D adopts 11 features for classification, and 3D adopts 8 and 11 features for classification, respectively, as shown in Table 4. For the multi-classification problem of abnormal gait behavior, we introduce the evaluation index Macro-average method and use Recall and Precision to express the classification results.

TP represents the number of samples that predict the correct gait as the correct gait; FN represents the number of samples that predict the correct gait as the incorrect gait; FP represents the number of samples that predict the incorrect gait as the correct gait; TN represents the number of samples that predict incorrect gait as incorrect gait;

(6) $$Precision=\frac{TP}{TP+FP}$$

(7) $$Recall=\frac{TP}{TP+FN}$$

(8) $$Precision_{\mathrm{macro}}=\frac{1}{n}\sum_{i=1}^{n}Precision_{i}$$

(9) $$Recall_{\mathrm{macro}}=\frac{1}{n}\sum_{i=1}^{n}Recall_{i}$$

**Figure 7:**
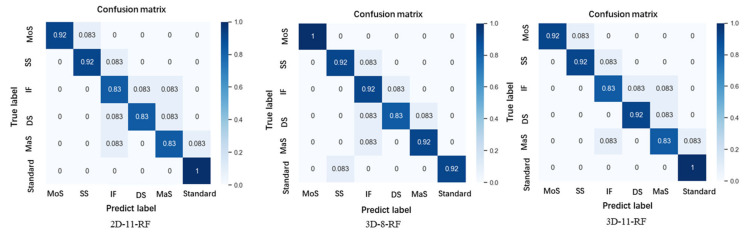
Best recognition precisions for 2D and 3D features.

In this experiment, we used five machine learning algorithms to classify the feature data of abnormal gait behavior. As seen in Table 3, the recognition accuracies were improved after the 3D reconstruction of some features extracted from the OP model, with the highest precisions being 89.18% for 2D features and 92.13% for 3D features. As shown in Figure 7, high recognition accuracy was achieved for all gaits using random forest (RF). The lower recognition accuracy of abnormal gait types also reached 83%, and the highest recognition can reach 100%. The different gait recognition accuracy of 3D features has been improved to varying degrees, indicating that abnormal gait features show more obvious differences after 3D reconstruction. The highest recognition accuracy for abnormal gait (Magnetic step) caused by Parkinson’s disease is 92%. Under the interference of a large number of different abnormal gaits, Parkinson’s gait achieved the same level of accuracy as previous work [1,2]. The overall experimental results were as expected, and high recognition accuracy was achieved for different types of abnormal gaits.

This work realizes the lightweight of the model and quickly completes the gait recognition of volunteers, which overcomes the problems caused by wearing sensors and include multiple types of abnormal gait diseases and is no longer limited to Parkinson’s [1]. However, with the introduction of a more abnormal gait, there may be some impact on Parkinson’s recognition.

## 5. Conclusions

In this paper, we presented a lightweight contactless pose sensing scheme for abnormal gait behavior recognition. With this scheme, a lightweight OP model was used to extract abnormal gait features in experiments and satisfactory results were achieved for the recognition of diseases with abnormal gait behavior. The scheme offered a more lightweight and less hardware-intensive alternative to traditional approaches for the recognition of abnormal behavior in the elderly. Specifically, we used Depthwise Separable Convolution to make the OP model more lightweight, with its FLOPs and Params reduced to 17.36% of the original model. This design reduced the hardware requirements for the model and allowed for real-time contactless recognition of abnormal gait behavior by cameras.

For the data collected by the OP model, we first performed 3D reconstruction on the lower limb data to obtain the real walking data. Then, we screened out the invalid features from the acquired features, completed feature importance analysis and filtered out gait features with poor results. Finally, we used five machine learning algorithms to classify the gait data and performed disease type recognition based on abnormal gait features. In the experiments, the RF algorithm achieved the best recognition precisions, which was 92.13%. The experiments verified that our proposed scheme can classify diseases with abnormal gait behavior accurately and efficiently. This scheme can assist doctors to recognize patient lesions by different abnormal gait behavior caused by different diseases. With this scheme, we can continue to study high-precision quantitative evaluation of such diseases in the future.

## Figures and Tables

**Table 1:** Characteristics of abnormal gait behavior for different diseases.

Gait	Gait Characteristics	Corresponding Types of Diseases
Magnetic step (or Freezing gait)	The walking steps are small and the movements are stiff and slow.	This gait may indicate Parkinson’s disease. The patient has symptoms of tremor, stiff limbs, and slow movement [20]
Mop step	The patient moves their left and right legs at inconsistent paces, and tends to walk by dragging their feet.	This gait may indicate lumbar disc herniation or cervical spondylitis myelopathy. Due to nerve compression, the patient has weak muscle on one leg, and generally drags one foot during walking [18]
Scissor Step	The patient tends to walk with their toes facing inward and their legs crossed.	This gait may indicate cerebral palsy or spinal cord injury, which can lead to impaired neurological function and affect physical activity [21]
Intermittent fragmentation	The patient experiences lameness and often feels the need to stop and rest due to pain and numbness in legs.	This gait may indicate osteoarthritis, lumbar spinal stenosis, vasculitis, or diabetes [22]
Drunk step	The patient cannot walk in a straight line and tend to stagger.	This gait may indicate cerebral hemorrhage, cerebral infarction, brain tumor, or cerebellar lesions. These diseases can cause cerebellar damage or cerebellar dysfunction [23].

**Table 2:** Acceptance scores for different numbers of features.

Number of Features	Score-2D	Score-3D
11	0.9167	0.9306
10	0.9028	0.8889
9	0.8889	0.9167
8	0.8472	0.9306
7	0.8611	0.8611
6	0.8472	0.8750
5	0.8056	0.8333
4	0.7639	0.6944
3	0.7083	0.7083
2	0.5556	0.5833
1	0.3333	0.4028

**Table 3:** Parameter combinations for machine learning models.

Machine Learning Algorithms	Parameters
GB	α = 10, loss function = deviance, subsample = 1.0.
KN	Weights = distance, *n* = 4, distance measure = 1.
MLP	Activation = ReLU, χ = (50,50), optimizer = Adam, α = 800, γ = 1.
RF	Number of decision trees = 57.
SVM	Kernel = ‘linear’, Kernel coefficient = 1.

*n* is the number of neighbors, α is the maximum number of iterations, γ is the state of the random number generator.

**Table 4:** Recognition results obtained for 8 and 11 features using five machine learning algorithms.

Machine Learning Algorithms	2D—11 Features		3D—8 Features		3D—11 Features	
	Recall	Precision	Recall	Precision	Recall	Precision
GB	0.7661	0.7778	0.8333	0.8611	0.8194	0.8472
KN	0.7211	0.7361	0.7500	0.7778	0.7533	0.7638
MLP	0.7557	0.7778	0.7944	0.8055	0.8344	0.8472
RF	0.8888	0.8918	0.9167	0.9213	0.9032	0.9048
SVM	0.7881	0.7918	0.8917	0.9027	0.8571	0.8611

## Data Availability

The data presented in this study are available in the link: https://github.com/dlj0214/GAIT (accessed on 4 May 2022).
